# Subjective well-being and month-long LF/HF ratio among deskworkers

**DOI:** 10.1371/journal.pone.0257062

**Published:** 2021-09-07

**Authors:** Kiko Shiga, Keisuke Izumi, Kazumichi Minato, Tatsuki Sugio, Michitaka Yoshimura, Momoko Kitazawa, Sayaka Hanashiro, Kelley Cortright, Shunya Kurokawa, Yuki Momota, Mitsuhiro Sado, Takashi Maeno, Toru Takebayashi, Masaru Mimura, Taishiro Kishimoto

**Affiliations:** 1 Department of Neuropsychiatry, Keio University School of Medicine, Tokyo, Japan; 2 Division of Rheumatology, Department of Internal Medicine, Keio University School of Medicine, Tokyo, Japan; 3 National Hospital Organization Tokyo Medical Center, Tokyo, Japan; 4 Medical AI Center, Keio University, Tokyo, Japan; 5 Faculty of Human Health, Aichi Toho University, Nagoya, Japan; 6 Center for Stress Research, Keio University, Tokyo, Japan; 7 Human System Design Laboratory, Graduate School of System Design and Management, Keio University, Kanagawa, Japan; 8 Department of Preventive Medicine and Public Health, Keio University School of Medicine, Tokyo, Japan; 9 Psychiatry Department, Donald and Barbara Zucker School of Medicine, New York, NY, United States of America; Chiba Daigaku, JAPAN

## Abstract

The importance of workers’ well-being has been recognized in recent years. The assessment of well-being has been subjective, and few studies have sought potential biomarkers of well-being to date. This study examined the relationship between well-being and the LF/HF ratio, an index of heart rate variability that reflects sympathetic and parasympathetic nerve activity. Pulse waves were measured using photoplethysmography through a web camera attached to the computer used by each participant. The participants were asked to measure their pulse waves while working for 4 weeks, and well-being was assessed using self-reported measures such as the Satisfaction With Life Scale (SWLS), the Positive and Negative Affect Schedule (PANAS), and the Flourishing Scale (FS). Each of the well-being scores were split into two groups according to the median value, and the LF/HF ratio during work, as well as the number of times an LF/HF ratio threshold was either exceeded or subceeded, were compared between the high and low SWLS, positive emotion, negative emotion, and FS groups. Furthermore, to examine the effects of the LF/HF ratio and demographic characteristics on well-being, a multiple regression analysis was conducted. Data were obtained from 169 participants. The results showed that the low FS group had a higher mean LF/HF ratio during work than the high FS group. No significant differences were seen between the high and low SWLS groups, the high and low positive emotion groups, or the high and low negative emotion groups. The multiple regression analysis showed that the mean LF/HF ratio during work affected the FS and SWLS scores, and the number of times the mean LF/HF ratio exceeded +3 SD had an effect on the positive emotion. No effect of the LF/HF ratio on negative emotions was shown. The LF/HF ratio might be applicable as an objective measure of well-being.

## Introduction

Well-being has been defined as a person’s overall state and is understood to be a multidimensional concept including affective reactions as well as cognitive judgments [1]. According to the Organization for Economic Co-operation and Development (OECD) guidelines, well-being is defined as “Good mental states, including all of the various evaluations, positive and negative, that people make of their lives and the affective reactions of people to their experiences.” Hence, well-being encompasses three elements: life evaluation (a reflective assessment of a person’s life or some specific aspect of it), affect (a person’s feelings or emotional states, typically measured with reference to a particular point in time), and eudaimonia (a sense of meaning and purpose in life, or good psychological functioning) [2]. Well-being has been shown to be related not only to mental health but also to physical health. Previous studies have suggested that people who report high levels of well-being live longer and also healthier lives than those with lower well-being [3–5].

In terms of employee well-being, previous research has shown that well-being is related to creativity and productivity [6], and employees with high well-being have 37% higher sales than lower employees [7]. Other studies have also found that well-being improves job performance [8–10], and employee well-being is related to presenteeism and absenteeism [11]. The general goal of the World Health Organization Mental Health Action Plan 2013–2020 emphasizes the importance of well-being: to promote mental well-being and to prevent mental disorders. The plan states that governments should put in place actions to protect and promote mental well-being at all stages of life [12]. As a result, there is a growing interest in the study of well-being around the world [13]. Naturally, well-being in the workplace has been receiving a lot of attention from the research community [14].

Many studies to date have sought relationships between objective biomarkers and psychological states and/or psychiatric disorders. For example, hypothalamic–pituitary–adrenal (HPA) axis dysregulation in patients with major depressive disorder (MDD) has been one of the most widely suggested pathophysiology and/or objective markers [14]. For anxiety disorders, studies have shown that patients with high anxiety had lower immunoglobulin A, lower melatonin, and higher alpha-amylase levels than those with low anxiety [15]. On the other hand, only a few reports have examined the relationship between well-being and biomarkers. Petra et al., in a study of 26 white-collar workers aged 24–62 years, found that that those with high well-being had significantly lower daily total cortisol and morning cortisol levels than those with low well-being. They also examined the association of well-being with mean daily blood pressure and the mean values of total catecholamine output, but no significant association was found [16]. Similar to Petra et al.’s study, Carol et al. reported in a study targeting 135 older women that those with higher levels of well-being started the day with lower cortisol levels and that their cortisol levels remained lower throughout the day, compared with subjects with lower levels of well-being [17].

Among other biomarkers, heart rate variability (HRV) has a high temporal resolution and represents one of the most promising quantitative markers of autonomic activity [18]. A relationship between HRV and depression has been reported. For example, patients with MDD have significantly lower RMSSD (root mean square of successive R-R differences) and SDNN (standard deviation of the NN intervals) values than healthy controls [19]. In addition, a study that compared HRV between patients with MDD and healthy controls found that there was no difference in heart rate between both subjects with remitted MDD and either subjects with current MDD or healthy controls. However, the current MDD group had a significantly lower mean SDNN, compared with the control group, whereas the remitted MDD group had a similar mean SDNN, compared with the healthy controls [20]. Although the potential usefulness of HRV as an indicator of psychological states has been suggested, there have been few reports on the relationship between HRV and well-being. Sloan et al. obtained electrocardiogram (ECG) recordings for 11 minutes in middle-aged and older Americans while they were answering questionnaires about well-being. Their results showed a negative correlation between negative emotions and HF values, but no association between HF values and positive emotions or psychological well-being (autonomy, environmental mastery, self-acceptance, personal growth, purpose in life, and positive relations with others) [21]. Andrew et al. also examined the relationship between well-being and heart rates during tasks in European-origin Caucasians between the ages of 45–59 years who were living in the London area. The results showed that men with low happiness had a higher mean heart rate during tasks than men with high happiness [22]. Geisler et al. tested the hypothesis that the emotion regulation strategies mediate the association between HRV and well-being. College students underwent 7 minutes HRV measurement when sitting still. The results showed that HRV was positively associated with positive hedonic tone (cheerfulness) and positive tense arousal (calmness), and these effects were mediated by the habitual use of executive emotion regulation strategies. Furthermore, although HRV was not correlated with present and expected future satisfaction with life, it was related with life satisfaction, again mediated by the habitual use of executive emotion regulation [23]. Thus, some significant relationships between HRV and well-being are reported to a certain degree, but it is not yet conclusive, and studies examining other populations or targeting a broader range of participants are warranted [5]. In addition, these studies of HRV measurements have been limited to resting and short-time records.

Among HRV, the Low Frequency (LF)/High Frequecny (HF) ratio is used to indicate the amount of sympathovagal modulation of the instantaneous heart rate [24, 25]: a low LF/HF ratio reflects parasympathetic dominance, while a high LF/HF ratio indicates sympathetic dominance [26–28]. A number of studies have been conducted using the LF/HF ratio to examine its association with psychological states, such as depression, stress, anxiety, fatigue, and burnout [29–35]. Yener et al. showed that the LF/HF ratio of surgeons was increased by 66 percent during surgery, compared with the baseline value [30]. In a study comparing patients with generalized anxiety disorder (GAD) and healthy controls, Chang et al. showed that patients with GAD had a significantly higher LF/HF ratio than controls at rest, and GAD patients exhibited a blunted LF/HF ratio reactivity to orthostasis [35]. On the other hand, few studies have examined the relationship between well-being and the LF/HF ratio. Bartczak et al. reported a study in which 10 patients with primary hypertension (including 4 patients with concomitant heart rate disturbances) underwent 24-hour ECG monitoring, but no correlation between the Satisfaction With Life Scale (SWLS) and the LH/FH ratio was observed [36].

Of note, in many of these previous studies, the relationship with the LF/HF ratio was mainly measured in experimental environments or for short time periods, such as 5 to 10 minutes. Some studies measured it for 24 to 48 hours, but their sample sizes were small. To examine whether the LF/HF ratio can be used as an indicator of daily stress or well-being, it may be important to measure the LF/HF ratio over a long period of time or under circumstances where the subject spends long periods of time, such as during work or at home. Therefore, the purpose of this study was to collect pulse wave data from workers continuously during work and to examine their LF/HF ratios and well-being. The contribution of this study was to examine whether new discoveries can be made about the association between well-being and LF/HF ratio by conducting long-term measurements, as opposed to laboratory measurements. We believe that the contactless measurement method was useful in the sense that it made it possible to measure with less burden on the participants. We hypothesized that workers with lower well-being would have higher mean LF/HF ratios during work, compared with workers with higher well-being; in other words, we expected workers with lower well-being to have a sympathetically dominant state. We are also interested in the fluctuation of the LF/HF ratio within individuals and hypothesized that workers with lower well-being might have greater fluctuation (i.e., the number of times the LF/HF ratio exceeds a certain threshold) than those with higher well-being.

## Materials and methods

### Study design

This study is part of a research project titled “Unobtrusive Sensing Technology for Quantifying Stress and Wellbeing to Promote a Healthy Workplace”. The concept, methodology, and overall goals of the study are described elsewhere [37]. The main purpose of this study was to examine the relationships among stress, well-being, and biological signals such as HRV, voice, and electrodermal activity from various perspectives. In this paper, we will focus on HRV and well-being. This study protocols have been registered with the University Hospital Medical Information Network (UMIN) (UMIN ID: UMIN000036814). This study was approved by the ethics committees of Keio University School of Medicine. All procedures performed in studies involving human participants were in accordance with the ethical standards of the institutional and/or national research committee, as well as with the 1964 Helsinki declaration and its later amendments or comparable ethical standards, and with Ethical Guidelines for Medical and Health Research Involving Human Subjects. Written informed consent was obtained from all participants. We collected pulse wave data during working hours from deskworkers for 4 weeks (20 working days). The participations provided their demographic characteristics such as age and sex at the beginning of the study and answered questionnaires on well-being throughout the 4-week period.

### Participants

The inclusion criteria for the participants in this study were adult workers (≥20 years old) mainly engaged in deskwork: specifically, a person who sits in front of a computer for at least half their working hours (3.5 hours a day or more). People who correspond to any of the following groups are excluded from this study:

1. People currently receiving treatment for mental illness, such as depression.
2. People who suffer from diseases that may affect the acquisition of biometric information. For example, those who have a disease or disorder that affects pulse wave data measurement (persons who have paralysis or involuntary movements on their faces, or persons with heart disease, etc.).
3. People who have difficulty operating a computer, such as using email or the internet.
4. People who cannot offer biometric information to researchers due to business/security reasons.

### Data collection

#### Demographic characteristics

Study participants were asked to provide demographic characteristics including age, sex, type of job, type of employment (regular employee/contract employee/part timer), years of employment, past medical history household income, etc., as well as lifestyle information, such as commute time, commute method, sleep hours on weekdays, sleep hours on holidays, smoking habits (smoking or non-smoking), and drinking habits (none/socially or habitually).

#### Self-rating scales related to well-being status

Study participants were given an e-mail with a URL link to the research website that provided self-report questionnaires about well-being; the participants were asked to complete the questionnaires online. Well-being was assessed using the SWLS [38], the Positive and Negative Affect Schedule (PANAS) [39], and the Flourishing Scale (FS) [40].

The SWLS is a self-reported questionnaire consisting of five questions. The questionnaire focusses specifically on assessing life satisfaction. The total score ranges from 5 to 35 points, with higher scores indicating higher satisfaction [41].

The PANAS is a questionnaire that measures positive and negative affect. There are 20 items in the questionnaire, with 10 items each for positive and negative affect [42]. Total scores range from 10 to 60 points each for positive and negative affect, respectively.

The FS consists of 8 items describing important aspects of human functioning, ranging from positive relationships to feelings of competence, meaning, and purpose in life [43]. Higher scores indicate that the respondents view themselves in positive terms in diverse areas of human functioning [40]. The questionnaire is answered using a 7-point scale, with a total score range of 8 to 56.

#### Sleep quality

Each business day, participants were sent an e-mail with a unique URL and asked to answer a questionnaire. For daily sleep quality, the Japanese version of the Athens Insomnia Scale (AIS-J) was used to gauge the quality of sleep based on a 4-point scale (1: Very dissatisfied, 2: Fairly dissatisfied, 3: Slightly dissatisfied, 4: Satisfied) [44]. For the analysis, the mean score of each individual was calculated and used.

#### Pulse wave data

Pulse waves were measured using a contactless vital sensing system developed by Connected Solutions Company Panasonic Corporation. This sensing system recognizes the facial image of a participant through a camera built into or connected to the computer and extracts pulse wave data using a photoelectric pulse wave method. A strong correlation in the R-R Interval values was found between the measurements obtained using the sensing system and portable electrocardiography (Check My Health, TRYTECH, Tokyo, Japan) (r^2^ = 0.978, *P* < 0.00001) [45]. The participants installed the system on their computer used in their workplace and measured their pulse waves during working hours. The sensing system was activated automatically as soon as the computer was started. The pulse wave data was automatically sent to cloud storage through the software.

If the R-R interval exceeded the mean value ± 3 SD, the interval was considered erroneous or nonstationary and was rejected [46]. HRV analyses in frequency domain are performed through spectral decomposition of the R-R interval signal using fast Fourier transform methods and then decomposed into the following frequency components in absolute power values (ms^2^): LF: 0.04–0.15 Hz, and HF: 0.15–0.4 Hz [28, 46, 47]. The LF and HF were estimated every 5 min, and the LF/HF ratio was calculated by dividing the power of the LF and HF bands [46].

### Data analysis

The distributions of all the variables were checked for normality using a histogram. The participants were split into low and high groups using the median values of the SWLS, PANAS, and FS scores.

First, a *t*-test was conducted to examine whether a significant difference in the mean LF/HF ratio during work was present between the high and low SWLS, positive emotion, negative emotion, and FS groups.

Second, to examine the fluctuation in the LF/HF ratio within an individual (i.e., the number of times the LF/HF ratio exceeded/subceeded a specific range of LF/HF ratios) during working hours was counted. Counts that exceeded a certain threshold were defined as follows: the number of counts of i) mean +0.5 SD, ii) mean +1 SD, iii) mean +2 SD, and iv) mean +3 SD during the study period. Counts that subceeded a certain threshold were defined as follows: the number of counts ⅴ) mean -0.5 SD, ⅵ) mean -1 SD, ⅶ) mean -2 SD, and ⅷ) mean -3 SD during the study period. To correct for changes in counts arising from differences in the measurement time across participants, the counts were divided by the total measurement time and the “counts per hour” was calculated. A *t*-test was conducted to examine whether there was a difference in the number of counts in i)-ⅷ) between the high and low SWLS, positive emotion, negative emotion, and FS groups.

Finally, a multiple regression analysis with a forward-backward stepwise selection method was conducted to examine the effects of the LF/HF ratio and demographic characteristics on SWLS, positive emotion, negative emotion, and FS. In Model 1, in order to simply examine the effect of the LF/HF ratio on well-being, a multiple regression analysis was conducted using the SWLS, positive emotion, negative emotion, and FS scores as dependent variables, and using the mean the LF/HF ratio during work, the number of times exceeding or subceeding a certain threshold, sex, and age as independent variables. In model 2, in order to exploratory examine the effect of wide range of demographic characteristics as well as the LF/HF ratio on well-being, a multiple regression analysis was conducted using the SWLS, positive emotion, negative emotion, and FS scores as dependent variables, and using the variables used in model 1 and demographic variables with p <0.2 in a univariate analysis (t-test and chi-squared) as independent variables. All the analyses were performed using the statistical package SPSS version 25.0 for Windows. Statistical significance was determined by p values of <0.05.

## Results

A total of 249 subjects from 11 Japanese companies participated in this study, but 80 participants were excluded because of missing questionnaire responses or pulse wave data records. Thus, the final sample comprised 169 participants. Comparing the 80 participants whose data were excluded from the analysis and 169 participants in the final sample, any demographic valuables, except the number of job changes, did not differ between groups (S1 Table). In addition, 6 of the 169 participants self-reported a history of hypertension.

The participants’ mean age (mean ± SD) was 39.2 ± 8.7 years, and 91 of the 169 participants (53.8%) were male; the mean length of employment ± SD was 9.3 ± 8.4 years (Table 1). The mean pulse wave measurement time ± SD was 81.9 ± 53.5 hours. The mean SWLS score was 21.1 ± 6.3, the mean PANAS positive emotion score was 34.8 ± 6.5, the mean PANAS negative emotion score was 28.0 ± 7.9, and the mean FS score was 39.1 ± 7.6.

**Table 1:** Participants’ demographic characteristics and relationship between SWLS, PANAS, FS, and demographic characteristics.

	Overall	SWLS			PANAS positive emotion			PANAS negative emotion			FS		
		Low group (<22.0)	High group (≥22.0)	*p* value	Low group (<35.0)	High group (≥35.0)	*p* value	Low group (<28.0)	High group (≥28.0)	*p* value	Low group (<39.0)	High group (≥39.0)	*p* value
	n = 169	n = 92	n = 77		n = 87	n = 82		n = 93	n = 76		n = 87	n = 82	
	M (±SD)	M (±SD)	M (±SD)		M (±SD)	M (±SD)		M (±SD)	M (±SD)		M (±SD)	M (±SD)	
	n (%)	n (%)	n (%)		n (%)	n (%)		n (%)	n (%)		n (%)	n (%)	
Age	39.23 (8.72)	37.70 (7.75)	41.06 (9.47)	0.014	38.95 (8.21)	39.52 (9.27)	0.673	40.55 (8.93)	37.62 (8.21)	0.029	38.64 (8.03)	39.85 (9.40)	0.369
Length of employment, in years	9.33 (8.40)	7.99 (6.92)	10.95 (9.70)	0.027	9.07 (7.47)	9.61 (9.32)	0.676	10.03 (9.12)	8.47 (7.38)	0.232	8.91 (6.90)	9.77 (9.75)	0.510
Working hours	9.19 (1.27)	9.08 (1.28)	9.32 (1.27)	0.212	9.22 (1.22)	9.16 (1.34)	0.775	9.18 (1.31)	9.20 (1.24)	0.922	9.34 (1.23)	9.03 (1.31)	0.123
Commute time, in min	52.07 (21.99)	52.42 (24.33)	51.64 (18.93)	0.820	54.02 (24.27)	49.98 (19.17)	0.234	51.34 (21.41)	52.96 (22.78)	0.635	55.72 (24.86)	48.15 (17.74)	0.025
PC usage, in h	7.54 (1.73)	7.64 (1.79)	7.42 (1.65)	0.407	7.72 (1.56)	7.35 (1.88)	0.181	7.44 (1.78)	7.66 (1.66)	0.426	7.63 (1.69)	7.44 (1.77)	0.480
Weekday sleep time, in h	6.13 (0.86)	6.03 (0.94)	6.25 (0.75)	0.089	6.15 (0.85)	6.11 (0.88)	0.766	6.20 (0.94)	6.05 (0.74)	0.241	5.98 (0.89)	6.29 (0.81)	0.021
Holiday sleep time, in h	7.49 (1.15)	7.44 (1.25)	7.56 (1.01)	0.505	7.52 (1.12)	7.46 (1.18)	0.737	7.36 (1.10)	7.66 (1.19)	0.093	7.51 (1.21)	7.48 (1.09)	0.892
Quality of daily sleep	3.20 (0.43)	3.11 (0.39)	3.32 (0.44)	**0.002**	3.13 (0.40)	3.28 (0.44)	0.024	3.30 (0.45)	3.09 (0.36)	**<0.001**	3.12 (0.41)	3.30 (0.42)	**0.005**
Male sex	91 (53.8)	51 (55.4)	40 (51.9)	0.651	40 (44.0)	52 (56.0)	0.035	52 (55.9)	39 (51.3)	0.551	53 (60.9)	38 (46.3)	0.057
Job type													
Managerial position	46 (27.7)	19 (21.3)	27 (35.1)	0.049	17 (19.8)	29 (36.3)	0.018	31 (33.7)	15 (20.3)	0.055	22 (26.2)	24 (29.3)	0.658
Other	120 (72.3)	70 (78.7)	50 (64.9)		69 (80.2)	51 (63.8)		61 (66.3)	59 (79.7)		62 (73.8)	58 (70.7)	
Full-time employee	147 (88.6)	82 (90.1)	65 (86.7)	0.488	77 (88.5)	70 (88.6)	0.984	78 (86.7)	69 (90.8)	0.406	78 (90.7)	69 (86.3)	0.368
Experience changing jobs	96 (56.8)	54 (58.7)	42 (54.5)	0.588	44 (50.6)	52 (63.4)	0.092	55 (59.1)	41 (53.9)	0.498	47 (54.0)	49 (59.8)	0.452
Discretionary work system	77 (47.2)	43 (48.9)	34 (45.3)	0.515	41 (47.7)	36 (46.8)	0.906	44 (49.4)	33 (44.6)	0.537	39 (47.0)	38 (47.5)	0.948
Telework is available	44 (26.7)	26 (28.6)	18 (24.3)	0.540	21 (25.0)	23 (28.4)	0.622	22 (24.2)	22 (29.7)	0.422	26 (30.6)	18 (22.5)	0.240
Commute method													
Train/Bus	152 (92.7)	79 (89.8)	73 (96.1)	0.124	80 (94.1)	72 (91.1)	0.464	82 (91.1)	70 (94.6)	0.349	75 (91.5)	77 (93.9)	0.549
Other	12 (7.3)	9 (10.2)	3 (3.9)		5 (5.9)	7 (8.9)		8 (8.9)	4 (5.4)		7 (8.5)	5 (6.1)	
Educational background													
High school/Junior college	21 (12.4)	15 (16.3)	6 (7.8)	0.095	13 (14.9)	8 (9.8)	0.307	15 (16.1)	6 (7.9)	0.106	12 (13.8)	9 (11.0)	0.579
University/More	148 (87.6)	77 (83.7)	71 (92.2)		74 (85.1)	74 (90.2)		78 (83.9)	70 (92.1)		75 (86.2)	73 (89.0)	
Smoking habit	38 (22.8)	23 (25.3)	15 (19.7)	0.395	26 (30.2)	12 (14.8)	0.018	21 (22.8)	17 (22.7)	0.980	22 (25.9)	16 (19.5)	0.326
Drinking habit	118 (70.2)	66 (71.7)	52 (68.4)	0.640	55 (63.2)	63 (77.8)	0.039	65 (70.7)	53 (69.7)	0.897	62 (52.5)	56 (47.5)	0.763
Household income													
2 million to <3 million yen	1 (0.6)	0 (0.0)	1 (1.4)	0.046	1 (1.3)	0 (0.0)	0.538	0 (0.0)	1 (1.4)	0.386	0 (0.0)	1 (1.4)	0.014
3 million to <5 million yen	19 (12.3)	13 (15.3)	6 (8.6)		11 (13.8)	8 (10.7)		13 (15.3)	6 (8.6)		12 (14.5)	7 (9.7)	
5 million to <7 million yen	21 (13.5)	16 (18.8)	5 (7.1)		13 (16.3)	8 (10.7)		9 (10.6)	12 (17.1)		17 (20.5)	4 (5.6)	
7 million to <10 million yen	46 (29.7)	26 (30.6)	20 (28.6)		24 (30.0)	22 (29.3)		24 (28.2)	22 (31.4)		26 (31.3)	20 (27.8)	
≥10 million yen	68 (43.9)	30 (35.3)	38 (54.3)		31 (38.8)	37 (49.7)		39 (45.9)	29 (41.4)		28 (33.7)	40 (55.6)	

Numbers in bold indicate that the differences did not disappear after Bonferroni correction.

There were no significant differences in demographic characteristics (age, sex, mean length of employment) between the high and low SWLS, positive emotion, negative emotion, or FS groups (Table 1). In terms of sleep quality, the high SWLS and FS groups had better sleep quality than the low SWLS and FS groups, respectively, and the low negative emotion group reported better sleep quality than the high negative emotion group. No significant difference in sleep quality was seen between the high and low positive emotion groups.

### Mean LF/HF ratio and well-being

The low FS group had a higher mean LF/HF ratio during work than the high FS group (Table 2). For SWLS, positive emotion, and negative emotion, no significant differences in the mean LF/HF ratio were seen between the high and low groups (Table 2).

**Table 2:** LF/HF ratio during work and fluctuation in the LF/HF ratio.

	SWLS				PANAS positive emotion				PANAS negative emotion				FS			
	Low group (<22.0)	High group (≥22.0)	t	*p* value	Low group (<35.0)	High group (≥35.0)	t	*p* value	Low group (<28.0)	High group (≥28.0)	t	*p* value	Low group (<39.0)	High group (≥39.0)	t	*p* value
	n = 92	n = 77			n = 87	n = 82			n = 93	n = 99			n = 87	n = 82		
	mean (± SD)	mean (± SD)			mean (± SD)	mean (± SD)			mean (± SD)	mean (± SD)			mean (± SD)	mean (± SD)		
Mean LF/HF ratio during work	0.572 (0.145)	0.585 (0.178)	0.560	0.576	0.563 (0.149)	0.567 (0.173)	-0.155	0.877	0.547 (0.165)	0.588 (0.153)	-1.668	0.097	0.594 (0.141)	0.535 (0.175)	2.386	0.018
Fluctuation of the LF/HF ratio (counts)																
Number of >mean +0.5 SD	259.7 (180.2)	244.8 (175.2)	0.543	0.588	264.1 (185.6)	241.1 (169.0)	0.843	0.401	262.0 (186.5)	241.8 (166.6)	0.734	0.464	257.8 (165.3)	247.8 (190.6)	0.365	0.715
Number of >mean +1 SD	149.6 (103.7)	143.3 (108.0)	0.386	0.700	153.7 (107.9)	139.3 (102.8)	0.883	0.378	150.6 (113.1)	142.0 (95.7)	0.529	0.598	148.5 (95.9)	144.8 (115.2)	0.229	0.819
Number of >mean +2 SD	36.3 (24.3)	35.0 (24.1)	0.354	0.724	38.2 (25.7)	33.0 (22.2)	1.391	0.166	36.6 (25.3)	34.6 (22.8)	0.512	0.610	37.8 (23.2)	33.5 (25.1)	1.172	0.243
Number of >mean +3 SD	7.0 (5.1)	7.8 (6.1)	-0.924	0.357	7.7 (5.8)	7.0 (5.3)	0.843	0.401	7.9 (6.3)	6.7 (4.5)	1.355	0.177	7.8 (4.5)	6.9 (6.5)	0.996	0.683
Number of <mean -0.5 SD	339.7 (218.7)	322.1 (213.6)	0.525	0.600	305.7 (228.4)	311.5 (201.2)	1.181	0.239	342.7 (227.3)	318.2 (201.7)	0.730	0.466	343.3 (301.0)	320.5 (231.4)	0.652	0.591
Number of <mean -1 SD	129.2 (92.0)	114.7 (88.7)	1.038	0.301	128.7 (96.5)	116.1 (83.8)	0.909	0.365	125.9 (93.4)	118.6 (87.4)	0.516	0.606	125.1 (83.6)	119.9 (97.8)	0.373	0.718
Number of <mean -2 SD	6.9 (4.3)	7.1 (6.8)	-0.279	0.780	6.3 (4.6)	7.7 (6.4)	-1.596	0.112	7.6 (6.6)	6.2 (3.9)	1.784	0.076	6.8 (4.5)	7.1 (6.6)	-0.343	0.290
Number of <mean -3 SD	1.5 (1.2)	1.5 (1.5)	0.082	0.935	1.4 (1.1)	1.6 (1.5)	-0.989	0.324	1.4 (1.1)	1.5 (1.5)	-0.731	0.466	1.4 (1.1)	1.5 (1.6)	-0.635	0.320

### Within-individual fluctuation of the LF/HF ratio and well-being

No significant differences in the number of times that the LF/HF ratio exceeded or subceeded a certain threshold were seen between the high and low SWLS, positive emotion, negative emotion, and FS groups (Table 2).

### Effect of the LF/HF ratio on well-being

In Model 1, variables related to the LF/HF ratio, sex, and age were included as independent variables. A high mean LF/HF ratio during work (β = -0.18) and a young age (β = 0.15) (R^2^ = 0.06) had an effect on a low SWLS score (Table 3). The number of times the mean LF/HF ratio exceeded +3 SD (β = -0.16) and a female sex (β = -0.20) (R^2^ = 0.06) had an effect on a low positive emotion score (Table 4). A young age (β = -0.24) (R^2^ = 0.06) had an effect on a high negative emotion score (Table 5). A high mean LF/HF ratio (β = -0.23) (R^2^ = 0.05) had an effect on a low FS score (Table 6).

**Table 3:** Effect of the LF/HF ratio on SWLS.

	SWLS							
	Model 1				Model 2			
	Unstandardized Coefficients		Standardized Coefficients	*p* value	Unstandardized Coefficients		Standardized Coefficients	*p* value
	B	SE B	β		B	SE B	β	
Mean LF/HF ratio during work	-6.81	2.95	-0.18	0.022	-7.33	2.82	-0.19	0.010
Age	0.11	0.05	0.15	0.046	-			
Quality of daily sleep	NA				5.38	1.09	0.37	<0.001
Household income	NA				1.44	0.44	0.24	0.001
R^2^	0.06				0.23			

B = Unstandardized regression coefficient, SE = Standard error, β = Standardized regression coefficient, R^2^ = coefficient of determination.

Model 1: LF/HF ratio during work, number of >mean +0.5 SD, number of >mean +1 SD, number of >mean +2 SD, number of >mean +3 SD, number of <mean -0.5 SD, number of <mean -1 SD, number of <mean -2 SD, number of <mean -3 SD, age, and sex.

Model 2 (SWLS): model 1 and length of service, weekday sleep time, quality of daily sleep, job type, commute method, educational background, and household income.

- = Used as an independent variable but not significant.

NA, not applicable.

**Table 4:** Effect of the LF/HF ratio on positive emotion.

	PANAS positive emotion							
	Model 1				Model 2			
	Unstandardized Coefficients		Standardized Coefficients	*p* value	Unstandardized Coefficients		Standardized Coefficients	*p* value
	B	SE B	β		B	SE B	β	
Number of > mean +3 SD	-0.19	0.09	-0.16	0.032	-0.19	0.09	-0.16	0.036
Sex (male = 0, female = 1)	-2.56	0.99	-0.20	0.010	-2.15	0.99	-0.17	0.031
Drinking habit	NA				2.37	1.07	0.17	0.028
Quality of daily sleep	NA				3.62	1.17	0.23	0.002
R^2^	0.06				0.13			

B = Unstandardized regression coefficient, SE = Standard error, β = Standardized regression coefficient, R^2^ = coefficient of determination.

Model 1: LF/HF ratio during work, number of >mean +0.5 SD, number of >mean +1 SD, number of >mean +2 SD, number of >mean +3 SD, number of <mean -0.5 SD, number of <mean -1 SD, number of <mean -2 SD, number of <mean -3 SD, age, and sex.

Model 2 (Positive emotion): model 1 and PC usage time, quality of daily sleep, job type, experience changing jobs, smoking habit, and drinking habit.

NA, not applicable.

**Table 5:** Effect of the LF/HF ratio on negative emotion.

	PANAS negative emotion							
	Model 1				Model 2			
	Unstandardized Coefficients		Standardized Coefficients	*p* value	Unstandardized Coefficients		Standardized Coefficients	*p* value
	B	SE B	β		B	SE B	β	
Age	-0.22	0.07	-0.24	0.002	-0.14	0.07	-0.16	0.043
Holiday sleep time	NA				1.14	0.55	0.16	0.038
Quality of daily sleep	NA				-3.79	1.39	-0.20	0.007
R^2^	0.06				0.11			

B = Unstandardized regression coefficient, SE = Standard error, β = Standardized regression coefficient, R^2^ = coefficient of determination. Model 1: LF/HF ratio during work, number of >mean +0.5 SD, number of >mean +1 SD, number of >mean +2 SD, number of >mean +3 SD, number of <mean -0.5 SD, number of <mean -1 SD, number of <mean -2 SD, number of <mean -3 SD, age, and sex.

Model 2 (Negative emotion): model 1 factors plus holiday sleep time, quality of daily sleep, job type, and educational background.

NA, not applicable.

**Table 6:** Effect of the LF/HF ratio on FS.

	FS							
	Model 1				Model 2			
	Unstandardized Coefficients		Standardized Coefficients	*p* value	Unstandardized Coefficients		Standardized Coefficients	*p* value
	B	SE B	β		B	SE B	β	
Mean LF/HF ratio during work	-10.80	3.57	-0.23	0.003	-10.72	3.35	-0.24	0.002
Quality of daily sleep	NA				5.57	1.29	0.32	<0.001
Household income	NA				1.33	0.52	0.19	0.011
R^2^	0.05				0.20			

B = Unstandardized regression coefficient, SE = Standard error, β = Standardized regression coefficient, R^2^ = coefficient of determination.

Model 1: LF/HF ratio during work, number of >mean +0.5 SD, number of >mean +1 SD, number of >mean +2 SD, number of >mean +3 SD, number of <mean -0.5 SD, number of <mean -1 SD, number of <mean -2 SD, number of <mean -3 SD, age, and sex.

Model 2 (FS): model 1 and working hours, commute time, weekday sleep time, quality of daily sleep, and household income.

NA, not applicable.

Next, as model 2, demographic characteristics were added to the above-mentioned variables related to the LF/HF ratio, sex, and age. Poor sleep quality (β = 0.37), low household income (β = 0.24), and a high mean LF/HF ratio at work (β = -0.19) (R^2^ = 0.23) had an effect on a low SWLS (Table 3). The number of times the mean LF/HF ratio exceeded +3 SD (β = -0.16), Poor sleep quality (β = 0.23), a female sex (β = -0.17), and a lack of drinking habits (β = 0.17) had an effect on a low positive emotion score (R^2^ = 0.13) (Table 4). A younger age (β = -0.16), the length of sleep hours on holidays (β = 0.16), and poor sleep quality (β = -0.20) had an effect on a high negative emotion score (R^2^ = 0.11) (Table 5). Poor sleep quality (β = 0.32), a high mean LF/HF ratio at work (β = -0.24), and a low household income (β = 0.19) had an effect on a low FS (R^2^ = 0.20) (Table 6). Including the presence of hypertension as an independent variable in the model 1 and 2, the results did not change.

## Discussion

Previous studies of the relationship between the LF/HF ratio and psychological states have mainly examined short-term measurements performed under experimental environments. Some studies have obtained measurements over 24 hours or 2 days, but such studies are still few in number and generally have had small sample sizes. The presently reported study is, by far, the largest to date to collect pulse wave data for longer than 24 hours to examine the relationship between the well-being of workers and the LF/HF ratio by continuously measuring pulse waves during work for 4 weeks. The results showed that the FS score was associated with the LF/HF ratio during work, suggesting that the LF/HF ratio might be useful as an objective measurement of well-being.

Although we hypothesized that a state of well-being would be correlated with the LF/HF ratio, we were only able to find that the FS, and not the SWLS or PANAS, was associated with the LF/HF ratio during work. The FS was developed by Diener et al. to measure psychological well-being, focusing especially on important aspects of human functioning ranging from having positive relationships to feelings of competence and having meaning and purpose in life [43]. Thus, participants with low levels of psychological well-being had a significantly higher LF/HF ratio than those with high levels, suggesting that they were in a sympathetic dominant state during work. To the best of our knowledge, the association between FS and the LF/HF ratio has not been previously studied. Several studies have examined the relationship between the LF/HF ratio and other psychological indicators, and these studies have shown that the LF/HF ratio was associated with stress, anxiety, overcommitment, and fatigue [29–35]. Because the FS reflects relationships with others and one’s feeling of contribution to society, it might have reflected the workers’ psychological status on the job more closely than the SWLS/PANAS.

As mentioned above, there were no significant differences in the LF/HF ratio between the high and low SWLS, negative emotion, and positive emotion groups. Regarding the SWLS group, only one study has examined a correlation between the SWLS and the LF/HF ratio in patients with primary hypertension; however, no significant association was found [36]. The present study is in line with this finding. On the other hand, the results of the multiple regression analysis showed that the LF/HF ratio during work had a significant effect on the SWLS after adjustments for demographic characteristics that may be associated with SWLS such as age and sex. However, due to the small coefficient of determination, caution should be exercised in interpretation. It was possible that variables that were not asked in this study had an effect on well-being. Therefore, large-scale studies that examine the interaction of more factors are needed.

Regarding negative and positive emotion, Sloan et al. showed that HF-HRV was not significantly related to any indices of negative emotion at the univariate level. However, HF had a negative effect on negative emotion when adjusted for covariates such as body mass index, menstrual status, and exercise. In contrast, positive emotion had no effect on HF in a univariate analysis and after adjustments for covariates [21]. Julian et al. found a meta-analysis of eight neuroimaging studies representing data from 191 participants demonstrating that brain regions including the amygdala and the medial prefrontal cortex that are involved in perceptions of threat and safety, which are characteristics more closely associated with negative rather than positive emotion, are also associated with HRV [48]. In this study, although it did not reach significant level, the high negative emotion group had a numerically higher LF/HF ratio during working hours than the low negative emotion group. On the other hand, a multiple regression analysis did not show an association between negative emotions and the LF/HF ratio. The main difference between the study by Sloan et al. and the presently reported study is that the age of the participants was 54.60 ± 11.55 in the former study, while the present study examined a younger group. Furthermore, the present study did not obtain data on body mass index, exercise, and other variables. The relationship between negative emotion and the LF/HF ratio may be clarified by further examining the relationships among various variables.

Focusing on individual fluctuations in the LF/HF ratio, we examined the relationship between well-being and the number of times that the LF/HF ratio exceeded or subceeded a certain threshold. The results showed no significant differences between the high and low SWLS, positive emotion, negative emotion, and FS groups. On the other hand, the results of the multiple regression analysis showed that the number of times the mean LF/HF ratio exceeded +3 SD had an effect on the positive emotion when considering the association with other factors such as demographic characteristics, although the effect was small. No effect of individual fluctuations in the LF/HF ratio on SWLS, FS, and negative emotion were shown. This result may indicate that regardless of a low- or high-level of well-being, many workers experienced a transition (rise and fall) in the LF/HF ratio during work. For well-being, a previous study suggested that the manner of spending one’s leisure time and one’s family relationships were important. For example, Li et al. showed that long working hours were related to a deterioration in well-being, but those who had leisure activities and hobbies had a higher well-being than those who do not, even if they worked long hours [49]. Huang et al. showed that work-family conflict (conflict between work life and family life) was negatively correlated with well-being [50]. Therefore, well-being may be affected not only by the conditions during work, but also by one’s way of spending time after work. This study only measured pulse waves during work, and this might be related to the absence of a significant difference in the LF/HF ratio between the high and low well-being groups.

Finally, the SWLS and FS are known to be relatively stable over time [51, 52] because their questionnaires ask questions about one’s life up until the time of the response. Interestingly, the LF/HF ratio during work was associated with FS and had an impact on SWLS, which is considered to remain unchanged for a long time, suggesting that one’s personal traits may be reflected in the HRV to a certain degree. In addition, for positive emotions, the individual fluctuations in the LF/HF ratio had an effect on positive emotion. The high peak of LH/FH expresses a state of tension and living a life that often forces one to be in a state of tension may make it difficult to feel positive. However, since the effect was small, caution is needed in interpretation.

The limitations of this study are as follows. The participants in this study were mainly deskworkers who worked using personal computers, which may limit the generalization of results to other occupations. This measurement was only performed for the time spent sitting and working at one’s computer during work, and this study was not able to measure the time at which the computer was turned off. In addition, it was possible that variables that were not asked in this study had an effect on well-being given the small coefficient of determination in multiple regression analyses. Furthermore, with regard to the bias of the sample, fewer older workers participated in the study, and the participants had a relatively high household income. Although the sample size of this study was larger than those of previous studies, targeting an even larger population with a wide range of demographic characteristics is warranted.

## Conclusions

This study examined the relationship between the LF/HF ratio and well-being by continuously measuring pulse waves during work. The results showed a significant association between the LF/HF ratio and FS during work. Further clarification of the relationship between well-being and HRV could lead to better assessments of well-being, which is currently assessed subjectively. Such assessments of well-being may be beneficial from the perspective of promoting health management and self-care at workplaces in the future.

## Supporting information

S1 Table Comparison of demographic characteristics of participants included and excluded in the analysis. (DOCX)
