# Supplementary material for: Subjective well-being and month-long LF/HF ratio among deskworkers
Source: PLoS One. 2021 Sep 7;16(9):e0257062. doi: 10.1371/journal.pone.0257062 (PMC8423311; doi:10.1371/journal.pone.0257062)
Supplement: S1 Table — (DOCX) [file pone.0257062.s001.docx]

S1 Table. Comparison of demographic characteristics of participants included and excluded in the analysis.

|  |  | | |
| --- | --- | --- | --- |
|  | Included group | Excluded group | *p* value |
|  | n = 169 | n = 80 |  |
|  | M (±SD)  n (%) | M (±SD)  n (%) |  |
| Age | 39.23 (8.72) | 36.66 (8.28) | 0.028 |
| Length of employment, in years | 9.33 (8.40) | 10.15 (7.94) | 0.469 |
| Working hours | 9.19 (1.27) | 9.29 (1.32) | 0.549 |
| Commute time, in min | 52.07 (21.99) | 48.97 (21.85) | 0.304 |
| PC usage, in h | 7.54 (1.73) | 7.33 (1.96) | 0.405 |
| Weekday sleep time, in h | 6.13 (0.86) | 5.89 (0.90) | 0.044 |
| Holiday sleep time, in h | 7.49 (1.15) | 7.46 (1.18) | 0.839 |
| Quality of daily sleep | 3.20 (0.43) | 3.06 (0.51) | 0.032 |
| Male sex | 91 (53.8) | 45 (56.3) | 0.722 |
| Job type |  |  |  |
| Managerial position | 46 (27.7) | 28 (36.4) | 0.173 |
| Other | 120 (72.3) | 49 (63.6) |  |
| Full-time employee | 147 (88.6) | 68 (85.0) | 0.431 |
| Experience changing jobs | 96 (56.8) | 27 (34.2) | **<0.001** |
| Discretionary work system | 77 (47.2) | 30 (41.1) | 0.381 |
| Telework is available | 44 (26.7) | 14 (17.7) | 0.125 |
| Commute method |  |  |  |
| Train/Bus | 152 (92.7) | 72 (94.7) | 0.553 |
| Other | 12 (7.3) | 4 (5.3) |  |
| Educational background |  |  |  |
| High school/Junior college | 21 (12.4) | 9 (11.8) | 0.897 |
| University/More | 148 (87.6) | 67 (88.2) |  |
| Smoking habit | 38 (22.8) | 27 (33.8) | 0.066 |
| Drinking habit | 118 (70.2) | 58 (73.4) | 0.607 |
| Household income |  |  |  |
| 2 million to <3 million yen | 1 (0.6) | 2 (3.2) | 0.028 |
| 3 million to <5 million yen | 19 (12.3) | 5 (8.1) |  |
| 5 million to <7 million yen | 21 (13.5) | 17 (27.4) |  |
| 7 million to <10 million yen | 46 (29.7) | 10 (16.1) |  |
| ≥10 million yen | 68 (43.9) | 28 (45.2) |  |

Numbers in bold indicate that the differences did not disappear after Bonferroni correction.
